# Factors associated with poor practice of dietary salt intake among patients with hypertension in a primary health care clinic in Malaysia

**DOI:** 10.1038/s41598-026-40124-2

**Published:** 2026-02-19

**Authors:** Yean Ken Ong, Siew Mooi Ching, Abdul Hadi Abdul Manap, Rozianita Mutazah

**Affiliations:** 1 Klinik Kesihatan Kampung Simee, Kementerian Kesihatan Malaysia, Jalan Kompleks Sukan, 31400 Ipoh, Perak Malaysia; 2 Department of Family Medicine, Faculty of Medicine and Health Science, Universiti Putra Malaysia, Serdang, Selangor Malaysia; 3 Malaysian Research Institute on Ageing, Faculty of Medicine and Health Sciences, Universiti Putra Malaysia, 43400 Serdang, Selangor Malaysia; 4 Sunway Medical School, Faculty ofMedical and Life Sciences, Sunway University, 5 Jalan Universiti, BandarSunway, 47500 Sunway City, Selangor Malaysia

**Keywords:** Hypertension, Nutrition

## Abstract

Excessive consumption of salt had contributed to uncontrolled blood pressure among hypertension patients in Malaysia. This study aims to assess the knowledge, attitude, and practice of dietary salt intake among hypertension patients, as well as identify factors that contribute to poor dietary salt intake habits. A cross-sectional study was carried out among hypertensive patients at Klinik Kesihatan Kampung Simee, Ipoh between January and March 2023. A systematic random sampling method was used to select participants. A self-administered questionnaire adapted from previous study was used. The questionnaire was then scored, and participants were categorized into group of poor and good knowledge, attitude, and practice of dietary salt intake. A total of 396 patients participated in the study, with a response rate of 92.5%. The median age of the study population was 63 years, and 54% of them were female. 49.5% of the participants were Chinese, followed by 32.3% Malay, 15.4% Indian, and 2.8% others. 65.2% had good knowledge on salt, 59.3% had good attitude towards salt reduction, but 31.3% had poor practice of dietary salt intake. According to the results of multiple logistic regression, Chinese ethnicity (AOR 3.055, 95%CI 1.419–6.577, *p* = 0.004), participants with ischemic heart disease (AOR 2.714, 95% CI 1.115–6.607, *p* = 0.028), poor knowledge of salt (AOR 1.802, 95%CI 1.114–2.916, *p* = 0.016), and poor attitude towards reducing salt (AOR 3.960, 95%CI 2.462–6.372, *p* < 0.001) were significantly associated with poor practice of dietary salt intake among participants. One in three hypertensive patients in this study failed to practise adequate salt restriction. Poor practice of dietary salt intake was significantly higher among Chinese participants, those with ischemic heart disease, individuals with inadequate knowledge or unfavourable attitude towards salt reduction. These findings underscore the importance of a targeted dietary counselling among different ethnicities with different culture and diverse traditional cuisines. The ministry of health should also intensified national salt reduction strategies with a stronger focus on closing knowledge gaps and reshaping attitudes. Future research should evaluate the effectiveness of salt reduction programmes among patients with non-communicable diseases.

**Supplementary Information:**

The online version contains supplementary material available at 10.1038/s41598-026-40124-2.

## Introduction

On a global scale, hypertension remains a major public health problem. However, only 21% of the patients attain the target blood pressure despite advances in antihypertensive therapies. Hypertension affects around 1.28 billion adults globally, of whom two-thirds live in low and middle-income countries; other regions have shown a reduction in mean systolic blood pressure over the last decade, but it has increased dramatically in Southeast Asia.^1^. In Malaysia, the prevalence of hypertension stands at 30%, with 6.4 million case reported, yet less than half (45%) of them achieve blood pressure control.^2^.

Excess in sodium intake had been identified as one of the contributing factors for elevated blood pressure and a higher risk of cardiovascular disease and all-cause mortality.^3^. A Cochrane review in 2020 showed that reducing sodium intake from 12.7 g to 6.05 g of salt resulted in a decrease in systolic and diastolic blood pressure by 7.75 mmHg and 2.68 mmHg, respectively among Asian hypertensive patients.^4^. In addition, lower sodium intake also enhances the effect of antihypertensive medication which will further help patient to control their blood pressure.^5^.

Despite the World Health Organisation’s (WHO) recommendation for a daily dietary salt intake of less than 5 g, Malaysians found to consumed an average of 7.9 g of salt daily.^6^. The same study also reported that Malaysians generally have poor practices related to dietary salt intake. This could be contributed by the diverse culture and food in Malaysia where most Malaysian consumed a lot of mee kolok, light soy sauce and curry noodle which is dense in salt.^6^. This is an alarming finding, as studies from high-income countries have also reported that most participants had poor knowledge, attitude and behaviors of dietary salt intake ^7^ despite ample evidence demonstrates the benefits of reducing dietary salt intake. Locally, there is a paucity of research addressing knowledge, attitude and practice particularly among patients with hypertension, a population susceptible to the adverse effects of high salt consumption.

In Malaysia, the highest prevalence of hypertension was recorded in the state of Perak (34%) as reported at a 1-in-3 affected adult, which is higher among other states in Malaysia. ^2^. In addition, rural populations always exhibiting higher rates of uncontrolled hypertension ^2^ Klinik Kesihatan Kampung Simee, which is located in a semi-urban part of the state serves a population density of appropriately 66 thousand, is an ideal setting in dealing with rising burden of cardiovascular diseases in the state through its hypertensive population given the state’s significant disease burden.^8^.

This study aims to address the gap in the levels of knowledge, attitude and practice related to dietary salt intake in the key population of hypertensive patients. This study also aims to identify the determinants of poor dietary practice which will help guide targeted interventions and support national salt reduction efforts among high-risk groups.

## Methodology

### Study design

A cross-sectional study was conducted among 428 hypertensive patients at Klinik Kesihatan Kampung Simee, Ipoh, Perak, Malaysia from January 1, 2023 to March 31, 2023. This clinic was selected due to Perak’s high hypertension prevalence (34%, the highest in Malaysia) and because Klinik Kesihatan Kampung Simee has one of the largest registered hypertension caseloads in the Kinta district, serving approximately 3349 patients.^2,9^.

Inclusion criteria were all hypertensive patients aged 18 years old and above and follow up under Klinik Kesihatan Kampung Simee for at least six months. Participants were able to understand English or Bahasa Malaysia. Exclusion criteria were pregnant women and patients with mental health conditions that impair consent or participation. The eligibility was verified through medical record review by the research team.

### Study population

All the hypertensive patients who attended follow up at Klinik Kesihatan Kampung Simee from 1st January 2023 until 31st March 2023 were included in the sampling frame. Then inclusion and exclusion criteria were checked by the researcher from the medical records before approaching the prospective participants for invitation to this study.

An explanation of the study including objectives, risk and outcome of the study was informed to participants. Those that agreed to participate will required to sign a written consent form.

### Sample size

The sample size was calculated using Lemeshow formula for two-sample proportion method, with a power of 80%, a significance level of 0.05 and 95% confidence level based on a study conducted by Hanbazaza et al. which reported 97.5%female patients had significantly higher salt knowledge score compared to male patients (90.9%) ^10^. The calculated sample size was 381 with a power of 80%, 95% confident interval and *p*-value set at 0.05. The final sample size was 476 after adjusted for 20% of non-response rate.^11^.

### Study instrument

The salt questionnaire contains knowledge, attitude and practices (KAP) sections. This KAP model aimed to investigate knowledge of health risks and advice; beliefs and intentions to undertake healthy behaviours in reducing sodium intake. This model has been used extensively in the nutrition literature to assess and identify gaps in health-related behaviors ^7,12–14^. The questionnaire was constructed by adjusting items from various existing sources (as indicated below).

- Knowledge Items: Items 1 to 8 were derived from the Malaysian Guideline for Healthcare Professionals ^14^. Item 9 on knowledge of certain high-sodium foods was modified from that of Sarmugam et al. ^12^.
- Attitude Items : Items 10 to 13 related to beliefs and intentions about reducing dietary salt intake, were based on those from the WHO/Pan American Health Organization (PAHO) salt questionnaire ^15^.
- Practice Items : Items 14 to 21 dealt with dietary and food-labeling behaviors, and were derived from a questionnaire developed by Khokhar et al. ^16^.

Translation and Validation : For linguistics and cultural concerns, the English version of the questionnaire were translated to Bahasa Malaysia and then back-translated to English by an independent translator as it was usually done for cross-cultural validation process. The last version was evaluated by a group of six experts (including one internist, four family physicians and one dietitian, all with at least 15 years of experience in the management of hypertension), respectively. The instrument had good content validity with a content validity index (CVI) of 0.98.

Each correct answer was awarded with 1 mark and the score was summed up according to the domains. The median score was selected as the cut-off point for categorizing ‘poor’ vs. ‘good’ practice as the total score was not normally distributed. This approach aligns with methods used in similar KAP studies assessing dietary salt intake practices, such as Algarni et al. for assessing practice on salt among medical students at King Abdulaziz University. ^17^ A pilot study with sample of 30 participants was conducted prior to the study and the reliability testing yielded Cronbach’s alpha values of 0.69 for knowledge, 0.81 for attitude, and 0.64 for practice. The value of Cronbach’s alpha for knowledge and practice was below the usual 0.7 which was the normally the accepted range but some researchers and studies suggest that a Cronbach’s alpha ranging from 0.64 to 0.85 is considered adequate.^18^.

### Data collection

A systematic random sampling was employed in this study. All the hypertensive patients registered under Klinik Kesihatan Kampung Simee were included into the sampling frame. The sampling interval (K) for the sample size was obtained by dividing estimated number of patients attending hypertension clinic in 3 months. The monthly attendance at the hypertension clinic were 800 patients, resulting in a total of 2400 hypertension patients over three months.

$$(k)=\frac{\text{total number of sample size}}{\text{total number of hypertension patients in }3\text{ months}}$$

The sampling fraction was calculated as 476 out of 2400, which was equivalent to 1:6. This implies that every sixth patient who attends the hypertension clinic was chosen and invited to participate in this study.

The first individual was selected using lottery method, by drawing lots. Subsequently every 6^th^ patient attending the clinic was selected using systematic random sampling according to the sampling interval (k) calculated as above. This cycle was repeated throughout the data collection period until the targeted number of study participants achieved. All participants were approached individually and a written consent was taken prior to data collection.

Participants were then given a set of questionnaires, they were required to answer part 1 and part 2 which was the demographic data and the questionnaire. After that, a research assistant will help to fill in the part 3 which consist of weight, height, blood pressure and the latest blood investigation based on participant’s medical record. Participants comorbidities were taken based on their medical records.

Finally, all the questionnaires were collected and was checked by the researcher assisted by the research assistant to check for missing data. All the missing data in the questionnaire were returned to the participants immediately and were filled up on the same day.

### Data analysis

The data were analysed using IBM Statistical Package for Social Science (SPSS) version 27 software. Categorical data were reported in frequencies and percentages, while continuous data were reported either mean and standard deviation (SD) or median and interquartile range (IQR) depending on the normality test result.

Association between poor salt practices and independent variables were examined using Chi-square test for categorical data and independent student t-test or Mann–Whitney U test for continuous data. Independent variables consisted of socio-demographic, clinical characteristic, knowledge of salt and attitude towards reducing salt.

Variables with a *p*-value < 0.25 in univariate analysis were entered into multiple logistic regression using a backward stepwise (likelihood ratio) method. The *P* < 0.25 was taken to avoid excluding any significant factors prematurely in the SLR as recommended by Hosmer and Lemeshow.^19^ All the assumptions were met where the dependent variable is dichotomous, no duplication of data, no multicollinearity detected, variance inflation factors was less than 10 and the collected sample size (n-396) had achieved the calculated sample size of 381.^20^The goodness of fit of the model was tested by Hosmer and Lemeshow test. A p-value of > 0.05 indicates the model was a good fit. The results for MLR are presented as adjusted odds ratios (OR) with 95% confidence interval (CI) and statistical significance was set at p < 0.05.

### Ethical approval

Our study had registered and obtained approval from the National Medical Research Register (NMRR) (ID 22–01,768-0M3). Ethical clearance was obtained from the Malaysian Medical Research and Ethics Committee (MREC) before the commencement of the study. Approval to carry out the study was also obtained from Jabatan Kesihatan Negeri Perak. All methods used in this research were performed in accordance with the relevant guidelines and regulations.

All participants were approached individually and a written informed consent was taken prior to data collection. Participants were given the option to decline the invitation. All information obtained from the study was treated with confidentiality and used only for this study. Data collected was only accessible by the principal investigator and will be kept in a locked cupboard in the principal investigator office for five years.

## Results

A total of 483 hypertensive patients were approached for the study. Among them, 428 patients fulfil the inclusion criteria as 55 of them didn’t fulfil the criteria mainly due to a lack of understanding of English or Bahasa Malaysia. Among 428 whom fulfilled the inclusion criteria, 32 patients declined to participate, citing reasons such as insufficient time to answer the questionnaires, lack of interest in the study and urgency to attend to waiting next of kin, making the response rate of 92.5% (396/428).

Table 1 shows the sociodemographic data of the study population. The median age of participants were 63 years old. More than half of the participants were female, and majority of the participants were of Chinese ethnicity. Most of the participants had highest secondary education.

**Table 1:** Sociodemographic of study population (n = 396).

Characteristic	Frequency (n)	Percentage (%)
Gender		
Female	214	54
Male	182	46
Age, years, median (IQR)	63	(55–70)
Ethnicity		
Malay	128	32.3
Chinese	196	49.5
Indian	61	15.4
Others	11	2.8
Marital status		
Single	42	10.6
Married	354	89.4
Monthly household income, RM		
< 4850	360	90.9
4850–10,959	29	7.3
> 10,960	7	1.8
Highest education level		
No formal education	9	2.3
Primary education	72	18.2
Secondary education	261	65.9
Tertiary education	54	13.6
Current employment status		
Public sector	33	8.3
Private sector	64	16.1
Self employed	47	11.9
Housewife	100	25.3
Unemployed	33	8.3
Retired	119	30.1

IQR, interquartile range.

Table 2 below shows the clinical characteristics as well as knowledge, attitude and practice of dietary salt intake of the participants. Over half of the study population had uncontrolled blood pressure and about 40.7% of them were obese. Despite the majority of participants demonstrated good knowledge and attitude towards reducing salt, there were still 31.3% having poor practice of dietary salt intake.

**Table 2:** Clinical characteristics, knowledge, attitude and practice of dietary salt intake of study population (n = 396).

Variables for clinical characteristics	Number, n	%
Body mass index BMI, kg/m^2^		
Underweight (< 18.5)	15	3.8
Normal (18.5 -22.9)	68	17.1
Pre-obese (23—27.4)	152	38.4
Obese (≥ 27.5)	161	40.7
Blood pressure in mmHg, median (IQR)	141/80	22/14
Duration of Hypertension in years		
1–9	223	56.3
≥ 10	173	43.7
Comorbidities, diabetes mellitus	148	37.4
Ischemic heart disease (IHD)	27	6.8
Dyslipidemia	289	73
Stroke	6	1.5
Chronic kidney disease	4	1
Smoking	50	12.6
Knowledge score, median (IQR)	5	2
Good	258	65.2
Poor	138	34.8
Attitude score, median (IQR)	3	1
Good	235	59.3
Poor	161	40.7
Practice, median (IQR)	5	3
Good	272	68.7
Poor	124	31.3

IQR, interquartile range; %, percentage.

Table 3 shows the association between poor practice and sociodemographic, clinical characteristic, knowledge as well as attitude of participants. From the bivariate analysis, those whom were of male gender, Chinese ethnicity, poor knowledge of salt score and poor attitude towards salt reduction were significant factors associated with poor practice of dietary salt intake.

**Table 3:** Association between sociodemographic, clinical characteristics, knowledge, attitude and poor practice of dietary salt intake using bivariate analysis (n = 396).

Variables	Practice of dietary salt intake		*P* value
	Good	Poor	
Age	63.0 (± 10.9)	64.0 (± 12.2)	0.499**
Gender			
Female	158 (73.8%)	56(26.2%)	0.017*
Male	114 (62.6%)	68 (36.4%)	
Ethnicity			
Malay	97 (75.8%)	31 (24.2%)	0.002*
Chinese	118 (60.2%)	78 (39.8%)	
Indian	50 (82%)	11 (18%)	
Others	7 (63.6%)	4 (36.4%)	
Marital status			
Married	244 (68.9%)	110(31.1%)	0.765*
Single	28 (66.7%)	14 (33.3%)	
Monthly income (RM)			
Less than 4850	245 (68.1%)	115 (31.9%)	0.364*
4851–10,959	23 (79.3%)	6 (20.7%)	
Above 10,960	4 (57.1%)	3 (42.9%)	
Education level			
Primary education	48 (59.3%)	33 (40.7%)	0.086*
Secondary	183 (70.1%)	78 (29.9%)	
Tertiary	41 (75.9%)	13 (24.1%)	
Employment			
Working and retirees)	195 (65.9%)	101 (34.1%)	0.038*
Housewife	77 (77%)	23 (23%)	
Body mass index BMI, kg/m^2^			
< 18.5	9 (60%)	6 (40%)	0.835*
18.5 to 22.9	45 (66.2%)	23 (33.8%)	
23 to 27.4	106 (69.7%)	46 (30.3%)	
≥ 27.5	112 (66.9%)	49 (30.4%)	
Blood pressure			
Controlled	122 (70.9%)	50 (29.1%)	0.399*
Uncontrolled	150 (67%)	74 (33%)	
Comorbidities			
Diabetes Mellitus, yes	104 (70.3%)	44 (29.7%)	0.600*
IHD, yes	13 (48.1)	14 (51.9%)	0.017*
Dyslipidemia, yes	193 (66.8%)	96 (33.2%)	0.179*
Smoking			
Nonsmoker	243 (70.2%)	103 (29.8%)	0.081*
Smoker	29 (58%)	21 (42%)	
Knowledge			
Good	199 (77.1%)	59 (22.9%)	< 0.001*
Poor	70 (50.7%)	68 (49.3%)	
Attitude			
Good	193 (82.1%)	42 (17.9%)	< 0.001*
Poor	79 (49.1%)	82 (50.9%)	

*Chi square test, **Mann Whitney U test IHD, ischaemic heart disease.

Table 4 identified four determinants of poor practice of dietary salt intake using multiple logistic regression. Poor attitude towards reducing dietary salt intake was the strongest predictors for poor practice of dietary salt intake among hypertensive patients. This was followed by those whom were of Chinese ethnicity. Hypertensive patients with ischemic heart disease had a 2.7 times higher risk of poor dietary salt intake practice compared to those who did not have ischemic heart disease and those with poor knowledge of salt were associated with higher odds of poor practice of dietary salt intake.

**Table 4:** Determinants of poor practice of dietary salt intake based on multiple logistic regression (n = 396).

Variables	aOR	95% CI, LL	UL	*p*-value
Gender				
Male	1.1	0.607	1.995	0.753
Female	1			
Ethnicity				
Chinese	3.055	1.419	6.577	**0.004**
Malay	1.967	0.86	4.5	0.109
Others	2.62	0.578	11.882	0.212
Indian	1			
Ischemic heart disease (IHD)				
Present	2.714	1.115	6.607	**0.028**
Absent	1			
Knowledge				
Poor	1.802	1.114	2.916	**0.016**
Good	1			
Attitude				
Poor	3.96	2.462	6.372	** < 0.001**
Good	1			
Education				
< Primary education	1.282	0.516	3.181	0.592
Secondary education	1.143	0.539	2.424	0.728
Tertiary education	1			
Employment				
Working and retirees	1.607	0.9	2.871	0.109
Housewife	1			
Smoking status				
Smoker	1.4	0.708	2.767	0.333
Not smoker	1			
Diabetes				
Present	0.919	0.806	1.047	0.205
Absent	1			

95%CI, 95% confidence interval; LL, lower limit; UL, upper limit aOR, adjusted odds ratio.

significant of bold when p is <0.05.

## Discussion

### Knowledge levels and gaps

Our study reported 65.2% of hypertension patient had good knowledge on salt. A similar high prevalence of good knowledge was also reported in other studies among general population in Malaysia, Saudi Arabia and Uruguay.^10,21,22^. This can be explained that these countries had adopted the WHO sodium reduction initiative that actively promotes education and awareness on salt reduction.^23^. However, misconceptions persist, as evidence by 57.8% of study population erroneously believing that drinking more water counteracts high dietary salt intake similar to finding by Chia et al.^24^. This misconception highlights a major gap in public knowledge about sodium and hypertension. Education campaigns should focus on correcting the false belief that drinking water can counteract excess salt, while emphasizing the importance of a low-salt diet. Besides that, 60.1% had poor knowledge on reading nutrition label among our study participants. The large number who found the labels difficult easily could indicate that current dietary counselling and public health messaging are not doing enough to guide patient how to use a nutrition label. Interventions should focus on the practical session on how to interpret ‘sodium’ on labels, which may be defined in mg, to a salt equivalent; ~ 2.5 g salt = 1 g sodium Existing MOH educational tools on label reading should be evaluated and revised in term of content and delivery methods. ^25^. An example is the recently implemented salt reduction in Finland where it was demonstrated that an individual must be taught on practical skills in order to affect behaviour change.^26^. Thus, consistent medical advice made a valuable contribution to increased knowledge and practice of low salt diet as well as achieve BP control among hypertensive patients. ^27^.

In this study, hypertension patients who had a poor knowledge were 1.8 times more likely to have poor practice of salt. Similar finding was reported in China where those with lesser knowledge score were associated with higher dietary salt intake.^28^. The possible explanation was that a person misconception on dietary intake may lead to poor dietary habits^25^. as more than half of the study population (57.8%) thought that drinking more water could reduce salt in the body. This misconception also had been shown to be significant factor associated with poorer knowledge on salt.^12^.

### Attitude towards salt reduction

In this study, 59.3% of hypertensive patients had a good attitude to salt reduction. Our prevalence was less than what has been reported by Haron et al. (75.5%) and Nadirah et al. (70.3%) ^22,29^; the discrepancy between these rates may be explained by our use of a more inclusive 4-item attitude measurement scale compared with 2-likert scale used in earlier studies. However, the fact that more patients have positive attitudes is in line with previous findings among Ethiopian hypertensive patients (66.4%) ^30^ and implies that overall, patients have a good understanding of their condition and are worried about limiting dietary salt intake ^31^.

But there’s one major obstacle from taking this can-do attitude to action: taste. Most of the patients concurred that low-salt diet is bland. This is also consistent with physiological evidence that salt taste threshold increases with age, such as a study among Japanese people reporting old subjects use higher salt concentrations for detection and consume more ^32^. This taste perception is a widely reported barrier to salt reduction among the general population ^33^.

We identified poor attitude as an independent predictor of poor practice, with negative attitudes increasing the risk of high dietary salt intake by nearly four times relative to positive attitudes. This finding is in agreement with similar reports from China, Lebanon and Australia ^34–36^. The discrepancy between attitude and behavior can be accounted for by a number of circumstances other than taste: Culinary traditions whose salty taste is a foundation of what constitutes good eating. Rigid social norms, including traditional cooking practices and communal meals that make personalized dietary adjustments hard. If the immediate pleasure of salty food trumps the more general, longer-term benefit of warding off these diseases, it could be a struggle to stick to salt-limited diets even with good intentions.

### Ethnic disparities in practice (Chinese ethnicity)

Our study reported that Chinese ethnicity was an independent risk factor for poor dietary salt intake practice because the odds of reporting a high level of dietary salt intake were threefold higher among Chinese in comparison to Indians. The result is consistent with the Malaysian Community Salt Survey (MyCoSS) in which Indian participants had better Knowledge, Attitude and Practice scores related to salt ^6,24^. Malaysia’s diverse ethnicity with various food culture plays a major role in shaping salt consumption patterns among different races. Malaysian Chinese cuisines in particular, their dishes rely heavily on sodium-rich components: light or dark soy sauce, fermented bean pastes, salted fish or preserved seafood, pickled vegetables and stock-based soups. In contrast, other ethnic groups rely more on spices, herbs, coconut milk rather than sodium-rich sauces. While the data on detailed sodium content of different races are limited, there were studies found that better salt-related practices among certain ethnic compared to Chinese.^6,24^.

The unique cuisine among different ethnicity in Malaysia requires a multifaceted approach from the food sector as well as policy makers to develop a lower-sodium version of traditional foods as well as to increase awareness among Chinese patients to cut down on their dietary salt intake.

### Ischemic heart disease (IHD) paradox: barriers to dietary adherence

Our study reported a concerning paradox. Participants with both hypertension and ischemic heart disease (IHD) had a 2.7 times higher risk of poor dietary salt intake compared to those with hypertension alone. This is consistent with a Japanese study in which patients with the historical diagnosis of CVD had higher dietary salt intake ^37^. Although the relationship between a high intake of salt and cardiovascular disease is well established ^3,38^, our finding underscores the evident mismatch between medical advice and actual practice.

This difference cannot be attributed to a lack of ability to adopt new lifelong habits. Instead, it is thought to signify a mixture of system and individual level barriers in the cardiac care continuum where the nutritional counseling as part of cardiac rehabilitation may be too general, or not personalized and hard to follow without continued encouragement and reinforcement. Another possible reason is adherence is tough due to denial of disease severity, poor perceived self-efficacy, and extent of taste alteration post-event can de-motivate individuals.

## Strength and limitation of the study

This study is one of the first study assessing knowledge, attitude and practice of dietary salt intake among hypertension patients in Malaysia. The findings from this study will help clinician identify those at risk of poor salt practice for further intervention. We encountered a couple of limitations. The current study is cross-sectional, and therefore no causal relationship can be presumed. Furthermore, we used self-reported questionnaire which may under-report the practice of the participant as they need to recall their practice as far as a month ago. The other reason is because they did not collect a 24-h urine sodium which is how he or she actually determine how much salt everyone ate. Certain participants might have indeed reported less dietary salt intake as there was no way to compare the same with any objective quantification of their salt intake. Another limitation was that the salt sensitivity was not assessed in this study, thus there is limitation affects the interpretation of certain findings. Hence, the results should be interpreted cautiously due to its limitations.

## Implications for policy and practice

The results from the study provide straightforward and actionable conclusions on potential further development of salt reduction efforts:

Personalized dietary counselling should be focused on high-risk hypertensive patients particularly Chinese ethnicity and those with ischemic heart disease. Counselling should be reinvented to focus on addressing practical barriers, including difficulties in interpreting nutrition labels and strong taste preferences for salty foods. This dietary counselling should be integrated into their regular chronic disease management especially for cardiac patients. Adoption of the more practical dietary counselling in the management of cardiac patients will provide a more comprehensive long-term disease management.

Rebranding public health campaigns: The MOH should scale-up national salt reduction programs and shift the focus away from general awareness. Efforts need to be hands-on and frequently address the misconceptions (eg, that drinking water erases the effects of salty foods) as well as provide practical skills so that the public, especially those most in need, such as reading and using nutrition labels for better food choices. This highlighted the important role of healthcare providers. Consistent brief advice, counselling, self-monitoring and education by trained clinicians, dieticians and other healthcare provider had been shown to significantly improved salt-related behaviours.^39^.

## Recommendations for future research

In order to expand on these results, the following research is recommended: Perform Randomized Controlled Trials (RCTs) with salt reduction interventions targeting high-risk patient groups, such as Chinese and IHD patients. Such trials need to clearly define the detail of intervention and use objective outcome measures, such as 24-h urinary sodium excretion for objectively monitoring adherence and effect. Besides, the contribution of salt sensitivity in this population, as such data may enhance clinical risk prediction and open the door for truly personalized dietary interventions.

## Conclusion

One in three hypertensive patients in this study failed to practise adequate salt restriction. Poor practice of dietary salt intake was significantly higher among Chinese participants, those with ischemic heart disease, individuals with inadequate knowledge or unfavourable attitude towards salt reduction. These findings underscore the importance of a targeted dietary counselling among different ethnicities with different culture and diverse traditional cuisines. The ministry of health should also intensified national salt reduction strategies with a stronger focus on closing knowledge gaps and reshaping attitudes. Future research should evaluate the effectiveness of salt reduction programmes among patients with non-communicable diseases.

## Supplementary Information

Below is the link to the electronic supplementary material.

Supplementary Material 1

## Data Availability

The datasets used and/or analysed during the current study available from the corresponding author on reasonable request.
