# Supplementary material for: Factors associated with poor practice of dietary salt intake among patients with hypertension in a primary health care clinic in Malaysia
Source: Sci Rep. 2026 Feb 19;16:9791. doi: 10.1038/s41598-026-40124-2 (PMC13013804; doi:10.1038/s41598-026-40124-2)
Supplement: Supplementary file 1 — Supplementary Material 1 [file 41598_2026_40124_MOESM1_ESM.docx]

Supplementary Table

Table S1 below reports the participants’ knowledge

Table S1: Knowledge of salt among study participants (n=396)

| No | Question | Answer (n=396) | | |
| --- | --- | --- | --- | --- |
|  |  | Yes (%) | No (%) | Don’t know(%) |
| 1 | Do you think high salt intake can cause serious health problem? | 339 (85.6) | 27 (6.8) | 30 (7.6) |
| 2 | Do you think that high salt intake could cause High blood pressure? | 344 (86.9) | 15 (3.8) | 37 (9.3) |
| 3 | Do you think that high salt intake could cause Stroke? | 251 (63.4) | 36 (9.1) | 109 (27.5) |
| 4 | Do you think that high salt intake could cause Heart disease? | 236 (59.6) | 45 (11.4) | 115 (29.0) |
| 5 | Do you think that high salt intake could cause Asthma? | 41 (10.4) | 143 (36.1) | 212 (53.5) |
| 6 | Do you think that high salt intake could cause kidney stones? | 254 (64.1) | 44 (11.1) | 98 (24.7) |
| 7 | Do you know the national recommended salt intake for an adult in a day? | 1 teaspoon  159 (40.2) | 1tablespoon  9 (2.3) | 228 (57.6) |
| 8 | Does Sodium/Natrium refers to salt in the Nutrition Information Panel? | 154 (38.9) | 9 (2.3) | 233 (58.8) |
| 9 | Do you think drinking more water will reduce salt in the body? | 229 (57.8) | 89 (22.5) | 78 (19.7) |

Table S2 below reports the participants’ attitude towards reducing salt

Table S2: Attitude towards reducing salt among study participants (n=396)

| No | Questions | Answers (n=396) | | | | |
| --- | --- | --- | --- | --- | --- | --- |
|  |  | Strongly disagree (%) | Disagree (%) | Neutral (%) | Agree (%) | Strongly agree (%) |
| 10 | Reducing salt to food is important to you? | 16 (4.0) | 21 (5.3) | 35 (8.8) | 247 (62.4) | 77 (19.4) |
| 11 | Food with low salt/ no salt is tasteless ? | 15 (3.8) | 84 (21.2) | 30 (7.6) | 234 (59.1) | 33 (8.3) |
| 12 | I try to eat a healthy diet | 14 (3.5) | 32 (8.1) | 30 (7.6) | 260 (65.7) | 60 (15.2) |
| 13 | You are concerned about the amount of salt in the daily diet? | 11(2.8) | 70 (17.7) | 75 (18.9) | 193 (48.7) | 47 (11.9) |

Table S3 below reports the participants’ practice of salt intake

Table S3: Practice of salt intake among study participants (n=396)

| No | Questions | Answer | |
| --- | --- | --- | --- |
|  |  | Yes (%) | No (%) |
| 14 | Do you add salt to the food at the table? | 22 (5.6) | 374 (94.4) |
| 15 | Do you add soy sauce to the food at the table? | 119 (30.1) | 277 (69.9) |
| 16 | Do you add extra salt in the cooking at home? | 14 (3.5) | 382 (96.5) |
| 17 | Do you avoid/minimize consumption of processed foods? | 319 (80.6) | 77 (19.4) |
| 18 | Do you look at the salt or sodium labels on food? | 113 (28.5) | 283 (71.5) |
| 19 | Do you use other spices to substitute salt when cooking? | 162 (40.9) | 234 (59.1) |
| 20 | Do you choose to buy food with low salt? | 236 (59.6) | 160 (40.4) |
| 21 | Do you avoid eating out? | 251 (63.4) | 145 (36.6) |
